# Feature-Based Molecular Networking to Target the Isolation of New Caffeic Acid Esters from Yacon (*Smallanthus sonchifolius*, Asteraceae)

**DOI:** 10.3390/metabo10100407

**Published:** 2020-10-13

**Authors:** Guillermo F. Padilla-González, Nicholas J. Sadgrove, Gari V. Ccana-Ccapatinta, Olga Leuner, Eloy Fernandez-Cusimamani

**Affiliations:** 1 Jodrell Laboratory, Royal Botanic Gardens, Kew, Kew Road, London TW9 3AB, UK; f.padilla@kew.org (G.F.P.-G.); n.sadgrove@kew.org (N.J.S.); 2 AsterBioChem Research Team, Laboratory of Pharmacognosy, School of Pharmaceutical Sciences of Ribeirão Preto, University of São Paulo, Av do café s/n, Ribeirão Preto 14040-903, SP, Brazil; cuscogarig@hotmail.com; 3 Department of Crop Sciences and Agroforestry, Faculty of Tropical AgriSciences, Czech University of Life Sciences Prague, Kamýcká 129, 16500 Prague, Czech Republic; leuner@ftz.czu.cz

**Keywords:** molecular networking, metabolomics, chlorogenic acids, caffeic acid esters

## Abstract

*Smallanthus sonchifolius* (yacon) is an edible tuberous Andean shrub that has been included in the diet of indigenous people since before recorded history. The nutraceutical and medicinal properties of yacon are widely recognized, especially for the improvement of hyperglycemic disorders. However, the chemical diversity of the main bioactive series of caffeic acid esters has not been explored in detail. In this metabolomics study, we applied the latest tools to facilitate the targeted isolation of new caffeic acid esters. Using liquid chromatography coupled to tandem mass spectrometry (LC-MS/MS), we analyzed extracts from different organs (roots, vascular tissues of the stems, stem epidermis, leaves, bracts, and ray flowers) and followed a feature-based molecular networking approach to characterize the structural diversity of caffeic acid esters and recognize new compounds. The analysis identified three potentially new metabolites, one of them confirmed by isolation and full spectroscopic/spectrometric assignment using nuclear magnetic resonance (NMR), high-resolution mass spectrometry (HRMS), and MS/MS. This metabolite (5-*O*-caffeoyl-2,7-anhydro-d-glycero-β-d-galacto-oct-2-ulopyranosonic acid), along with eight known caffeic acid esters, was isolated from the roots and stems. Furthermore, based on detailed tandem MS analyses, we suggest that the two isomeric monocaffeoyl-2,7-anhydro-2-octulopyranosonic acids found in yacon can be reliably distinguished based on their characteristic MS^2^ and MS^3^ spectra. The outcome of the current study confirms the utility of feature-based molecular networking as a tool for targeted isolation of previously undescribed metabolites and reveals the full diversity of potentially bioactive metabolites from *S. sonchifolius*.

## 1. Introduction

The South American Andes may be considered as the “cradle” of a surprisingly wide variety of edible tubers and roots, many of which have been consumed by native Andean people since before recorded history [1]. Apart from the introduced potato (*Solanum tuberosum* L, Solanaceae), Andean tuber crops have high nutraceutical and medicinal potential but have been neglected in recent times. These less-known crops include ahipa (*Pachyrhizus ahipa* (Wedd.) Parodi, Fabaceae), oca (*Oxalis tuberosa* Molina, Oxalidaceae), ulluco (*Ullucus tuberosus* Caldas, Basellaceae), and yacon (*Smallanthus sonchifolius* (Poepp. et Endl.) H. Robinson, Asteraceae) [1]. Fortunately, the demand for yacon has experienced a reawakening in light of the health benefits that have been described in recent decades. This species is especially popular for its ability to attenuate hyperglycemic disorders such as insulin resistance, metabolic syndrome, and diabetes [2,3,4]. Therapeutic effects of yacon are related to the abundance of fructooligosaccharides (FOSs) of the inulin type, which accumulate in the tuberous roots. These nonstarch soluble fibers inhibit digestive enzymes and slow the release of sugars from starches, which lowers the glycemic index of yacon and other starchy foods consumed concomitantly [4,5,6].

However, the hypoglycemic and antidiabetic effect of yacon is also modulated by specialized metabolites belonging to the class of polyphenols [7,8]. The major polyphenolic compounds in *S. sonchifolius* are caffeic acid derivatives that vary according to esterification sequences on quinic acid (also known as chlorogenic acids), altraric acid, and 2,7-anhydro-2-octulopyranosonic acid [9,10]. Caffeoylaltraric acids (CAAs) are a structurally diverse chemical class that is strongly represented in yacon, but only a few of them (i.e., 2,3,5- or 2,4,5-tricaffeoylaltraric acid) are present in large quantities in the roots [9]. Several studies have correlated the presence of these caffeic acid esters in yacon roots with its hypoglycemic properties. As an example, 2,3,5- or 2,4,5-tricaffeoylaltraric acid, isolated from yacon roots, has been patented as an agent that normalizes elevated blood glucose levels and also for use as a functional food additive [11]. 

However, the structural diversity of caffeic acid esters has not been explored in detail. Recent metabolomic studies suggest there is a wide diversity of caffeic acid esters in the different organs of yacon, although most of these accumulate in the roots [12]. For instance, several potentially new CAAs and caffeoyloctulosonic acids (COAs) have been observed in recent studies but were not characterized [12,13,14]. CAAs and COAs constitute two especially relevant chemical classes due to their medicinal properties and restricted distribution. COAs and their derivatives have been rarely reported from natural sources. Apart from yacon, COAs and a few analogous compounds were identified in extracts of *Erigeron breviscapus* by liquid chromatography coupled to tandem mass spectrometry (LC-MS/MS), but their absolute configuration has not been determined [15,16]. Although the presence of additional caffeoyloctulosonic acid derivatives [13] and undescribed caffeoylaltraric acids has been recently suggested in yacon [12], their chemical structures are not yet formally characterized.

Considering that recent developments in analytical techniques and computational tools have allowed a deeper exploration into natural product chemical diversity [17], in the current study, we aim to apply feature-based molecular networking (FBMN) to target the isolation of new caffeic acid esters from yacon. FBMN represents a recent computational tool that enables fast and semiautomated semiquantitative analysis of multiple LC-MS/MS files while allowing isomer resolution [18]. LC-MS/MS analyses and FBMN of yacon extracts from different organs cluster unknown metabolites according to class, greatly narrowing the search for new caffeic acid esters and facilitating our understanding of their distribution across plant organs. Further isolation and structural elucidation of selected mass features gave one new caffeic acid ester, in addition to eight known compounds. Thus, using a modern metabolomics tool (FBMN), we were able to efficiently explore the structural diversity and distribution of a specific class of specialized metabolites in yacon in high detail. Furthermore, the current study reiterates that *S. sonchifolius* represents a rich source of caffeic acid esters, including several hitherto undescribed metabolites. 

## 2. Results and Discussion

### 2.1. Feature-Based Molecular Networking of Different Organs

**Figure 1:**
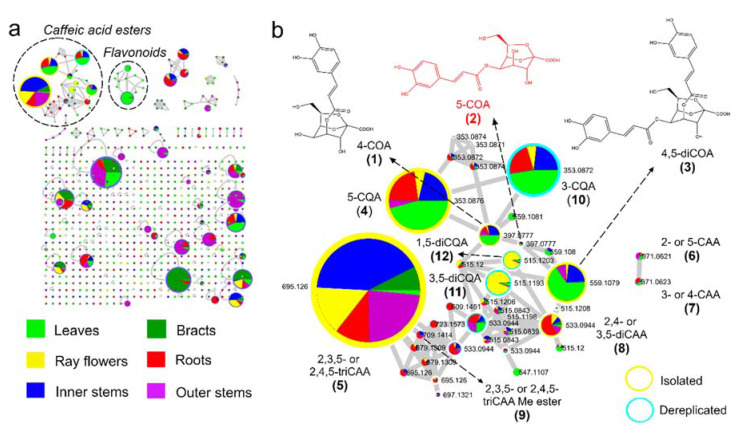
Feature-based molecular networking of different organs of yacon analyzed by ultra-high-performance liquid chromatography coupled to UV detection and high-resolution tandem mass spectrometry (UHPLC-UV-HRMS/MS) in negative ionization mode. (**a**) Entire molecular network, showing that caffeic acid esters group the higher number of nodes. Node size represents semiquantitative differences in metabolites concentrations in each plant extract, while node colors relate to the presence of each metabolite in different organs. (**b**) Amplified nodes of caffeic acid esters. Identity of metabolites as follows: 4-*O*-caffeoyl-2,7-anhydro-d-glycero-β-d-galacto-oct-2-ulopyranosonic acid, 4-COA (**1**); 5-*O*-caffeoyl-2,7-anhydro-d-glycero-β-d-galacto-oct-2-ulopyranosonic acid, 5-COA (**2**); 4,5-di-*O*-caffeoyl-2,7-anhydro-d-glycero-β-d-galacto-oct-2-ulopyranosonic acid, 4,5-diCOA (**3**); 5-*O*-(*E*)-caffeoylquinic acid, 5-CQA (**4**); 2,3,5- or 2,4,5-tricaffeoylaltraric acid, triCAA (**5**); 2- or 5-caffeoylaltraric acid, CAA (**6**); 3- or 4-caffeoylaltraric acid, CAA (**7**); 2,4- or 2,5-dicaffeoylaltraric acid, diCAA (**8**); 2,3,5- or 2,4,5-tricaffeoylaltraric acid methyl ester (**9**); 3-*O*-(*E*)-caffeoylquinic acid, 3-CQA (**10**); 3,5-di-*O*-(*E*)-caffeoylquinic acid, 1,5-CQA (**11**); 1,5-di-*O*-(*E*)-caffeoylquinic acid, 3,5-CQA (**12**). New metabolite highlighted in red. The identification of metabolites was done by isolation and interpretation of NMR and MS data (node edge yellow) or by analysis of MS data of the crude extract (node edge blue).

The metabolic profiles of methanol (80%) extracts from the leaves, stems epidermis, stems vascular tissues (inner stems), rhizomes, bracts, and ray flowers of *Smallanthus sonchifolius* were recorded by ultra-high-performance liquid chromatography coupled to UV detection and high-resolution tandem mass spectrometry (UHPLC-UV-HRMS/MS). Mass spectrometry data recorded in the negative ionization mode were further processed, as described in the Materials and Methods section, and submitted to feature-based molecular networking (FBMN) on the Global Natural Product Social Molecular Networking (GNPS) platform. Spectral library annotation and manual inspection of raw MS data revealed a clustering tendency by chemical class, where caffeic acid esters and flavonoids clustered the higher number of nodes (Figure 1). This analysis, along with database searches and compound isolation, facilitated the identification of the majority of previously reported caffeic acid esters in yacon [9,10], while demonstrating organ-specificity for all of them (Figure 1). 

Caffeic acid esters have been widely reported in *S. sonchifolius* [9,10] as well as in several members of the Asteraceae family, including species closely related to the genus *Smallanthus* [19,20]. However, recent mass spectrometry techniques and computational tools have created the opportunity for a re-exploration of the chemical diversity in multiple tissues, organs, and species in a fast and semiautomated way [17,18,21]. This has accelerated the discovery of new molecules by focusing on the isolation of undescribed molecules, avoiding the reisolation of known compounds if so desired. In this context, FBMN represents a powerful tool as it clusters structurally related molecules with similar MS^2^ spectra while differentiating isomeric metabolites eluting at different retention times [18]. As an additional advantage, this technique also captures semiquantitative information related to ion abundance.

In the case of ESI mass spectrometry, ion abundance represents both quantitative information and ionization efficiency. While the latter has the capacity to make quantitative estimation tedious when comparing compounds of the same chemical class under standardized conditions, the comparison of ion abundances can provide reliable data on the relative amount of metabolites. Hence, our data reliably conveys that caffeic acid esters are detected in all organs of yacon but accumulate at different ratios (Figure 1). For example, relatively higher amounts of monocaffeoylquinic acids (5-*O*-(*E*)-caffeoylquinic acid (**4**) and 3-*O*-(*E*)-caffeoylquinic acid (**10**); Figure 1) were detected in yacon leaves, while two dicaffeoylquinic acid isomers, namely, 3,5-di-*O*-(*E*)-caffeoylquinic acid (**11**) and 1,5-di-*O*-(*E*)-caffeoylquinic acid (**12**), were mainly detected in the ray flowers. These three compounds have been reported in different organs of yacon in previous studies [12]. Similarly, caffeoyloctulosonic acids (Compounds **1–3**) were accumulated in relatively higher amounts in the leaves, while yacon’s main caffeic acid ester, 2,3,5- or 2,4,5-tricaffeoylaltraric acid (**5**), was dominant in the vascular tissues of stems (Figure 1). Previous studies have reported high amounts of this compound in the roots and stems of yacon [12]. Interestingly, the presence of high amounts of 1,5- and 3,5-dicaffeoylquinic acid in the ray flowers of yacon (confirmed by UV detection) and 2,3,5- or 2,4,5-tricaffeoylaltraric acid is described here for the first time, but their ecological implications remain unknown.

**Figure 2:**
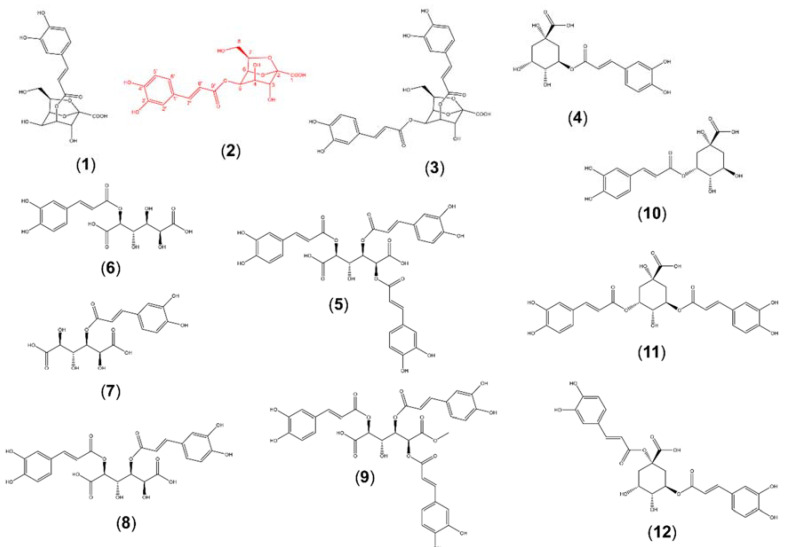
Chemical structures of the caffeic acid esters identified in yacon. 4-*O*-caffeoyl-2,7-anhydro-d-glycero-β-d-galacto-oct-2-ulopyranosonic acid, 4-COA (**1**); 5-*O*-caffeoyl-2,7-anhydro-d-glycero-β-d-galacto-oct-2-ulopyranosonic acid, 5-COA (**2**); 4,5-di-*O*-caffeoyl-2,7-anhydro-d-glycero-β-d-galacto-oct-2-ulopyranosonic acid, 4,5-diCOA (**3**); 5-*O*-(*E*)-caffeoylquinic acid, 5-CQA (**4**); 2,3,5- or 2,4,5-tricaffeoylaltraric acid, triCAA (**5**); 2- or 5-caffeoylaltraric acid, CAA (**6**); 3- or 4-caffeoylaltraric acid, CAA (**7**); 2,4- or 3,5-dicaffeoylaltraric acid, diCAA (**8**); 2,3,5- or 2,4,5-tricaffeoylaltraric acid methyl ester (**9**); 3-*O*-(*E*)-caffeoylquinic acid, 3-CQA (**10**); 3,5-di-*O*-(*E*)-caffeoylquinic acid, 1,5-CQA (**11**); 1,5-di-*O*-(*E*)-caffeoylquinic acid, 3,5-CQA (**12**). New metabolite highlighted in red.

In previous studies, we applied traditional molecular networking to study the metabolite diversity present in different organs of yacon [12] and to explore the association between the metabolic profiles of yacon leaves with the plant’s developmental stage and environmental variables [14]. These former analyses, however, did not lead to the identification of isomeric caffeic acid esters, nor did they provide quantitative information. However, by applying FBMN to a set of yacon extracts from different organs, we observed several isomers of caffeoylquinic, caffeoylaltraric, and caffeoyloctulosonic acids with different accumulation patterns, including eleven known compounds and one new substance (Figure 1 and Figure 2). As previously mentioned, nine metabolites were isolated and structurally characterized by combined MS and NMR spectroscopy to demonstrate agreement to stand-alone MS assignments (see Section 2.2). Our FBMN approach highlighted the presence of several undescribed molecules (i.e., *m*/*z* 397.077, 679.1309, and 723.1573; Figure 1) and guided the isolation and eventual identification of one new caffeic acid ester (5-*O*-caffeoyl-2,7-anhydro-d-glycero-β-d-galacto-oct-2-ulopyranosonic acid, compound **2**) and another compound that represents a new report for the genus (2,3,5- or 2,4,5-tricaffeoylaltraric acid methyl ester, compounds **9**; Figure 1). The structures of both metabolites were fully elucidated by HRMS, MS/MS, and 1D/2D NMR spectroscopy (see Section 2.2). 

### 2.2. Isolation and Structural Elucidation of Caffeic Acid Esters from Yacon

Considering that our analysis by FBMN and the dereplication approach suggested the presence of several potentially new metabolites, including one isomer of monocaffeoyloctulosonic acid (*m/z* 397.077; Figure 1), two structural analogs of 2,3,5- or 2,4,5-tricaffeoylaltraric acid (*m/z* 679.1309 and 723.1573; Figure 1), and a possible new report for the genus (*m/z* 709.1414; Figure 1), target isolation of those compounds was performed. A sample of freeze-dried methanolic extracts obtained from the vascular tissues of the stems (10 g) and roots (10 g) of yacon was submitted to classic isolation processes using Sephadex LH-20 column chromatography, followed by semipreparative HPLC (see Materials and Methods section), to afford nine pure compounds (compounds **1**–**9**; Figure 2). Furthermore, the tentative structures of three caffeoylquinic acids (compounds **10**–**12**; Figure 2) were suggested based on the analysis of MS^2^ data of crude extracts and its comparison with spectral data from the published literature [22]. 

Compounds **1**–**3** were identified as caffeoyloctulosonic acid derivatives based on HRMS, MS/MS, and NMR data (Table 1). Compound **2** was assigned as a new caffeoyloctulosonic acid derivative. The HRMS of **1** and **2** showed both a deprotonated molecule [M − H]^−^ at *m/z* 397.07745 (Figure S5), consistent with the molecular formula of C_17_H_18_O_11_ (calculated for C_17_H_17_O_11_, 397.07709), indicating these compounds are likely isomers. Their online UV spectra displayed absorbance maxima at 248, 300 (shd), and 330 nm, characteristic of phenylpropanoid derivatives. The ^1^H and ^13^C NMR spectrum of **2** (Table 1) resembled that of the previously reported 4-*O*-caffeoyl-2,7-anhydro-d-glycero-β-d-galacto-oct-2-ulopyranosonic acid [10] (compound **1**; Figure 2). However, the ^1^H and ^13^C NMR data (Figures S1 and S2), as well as HMBC correlations, suggested a different esterification position of the caffeoyl moiety in compound **2** (C5; Table 1) compared to compound **1** (C4) [10]. Relative to the ^1^H spectrum of compound **1**, diagnostic proton shifts of compound **2** include the downfield shift of the ^1^H doublet of doublets on C5 from 4.34 to 5.30 ppm, which is the position of esterification on compound **2**, and the upfield shift of the ^1^H doublet on C4, from 5.25 to 4.16 ppm, which is not esterified on compound **2** as it is on compound **1**. In conjunction with the HMBC correlation of the caffeoyl carbonyl carbon at 167.9 ppm to the octulosonic acid proton at 5.30 ppm, the data indicate that compound **2** is a new caffeoyloctulosonic acid derivative, assigned as 5-*O*-caffeoyl-2,7-anhydro-d-glycero-β-d-galacto-oct-2-ulopyranosonic acid. Due to the low yield of pure compound **2**, comprehensive HMBC correlations could only be seen on the mixture of compounds **1** and **2** (Figure S4), which demonstrated the position of caffeoyl esterification for both compounds.

Although it is possible that both **1** and **2** can be created by partial hydrolysis of compound **3**, their presence was verified by an LC-MS analysis of yacon leaves collected in liquid nitrogen to quench metabolism. HRMS, MS^2^, and NMR data of compound **3** indicated the presence of two caffeoyl moieties esterified to a 2,7-anhydro-d-glycero-β-d-galacto-oct-2-ulopyranosonic acid molecule, showing consistent values with a previously reported dicaffeoyloctulosonic acid derivative (4,5-di-*O*-caffeoyl-2,7-anhydro-d-glycero-β-d-galacto-oct-2-ulopyranosonic acid) isolated from yacon roots [10] (Figure 2).

The presence of different isomers of caffeoyl-2,7-anhydro-2-octulopyranosonic acid has been previously reported only in yacon roots [13] and *Erigeron breviscapus* (Asteraceae) [15,16] based on LC-MS/MS analyses. For instance, at least three different isomers of monocaffeoyl-2,7-anhydro-2-octulopyranosonic, including 5-*O*-caffeoyl-2,7-anhydro-d-glycero-β-d-galacto-oct-2-ulopyranosonic acid (compound **2**), were tentatively annotated in an earlier study of *E. breviscapus* [16]. However, due to the lack of direct comparisons with pure substances or detailed studies on the fragmentation patterns of isomeric caffeoyloctulosonic acids, the unambiguous identity of those compounds remained uncharacterized. 

**Figure 3:**
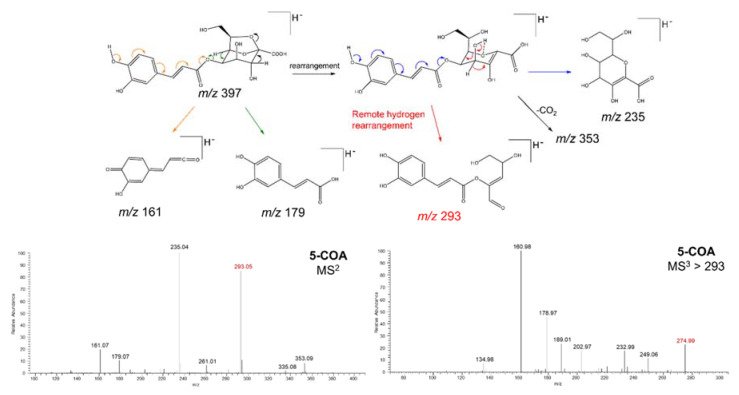
Proposed fragmentation pattern of compound **2** and its mass spectra (MS^2^ and MS^3^) in the negative ionization mode.

**Figure 4:**
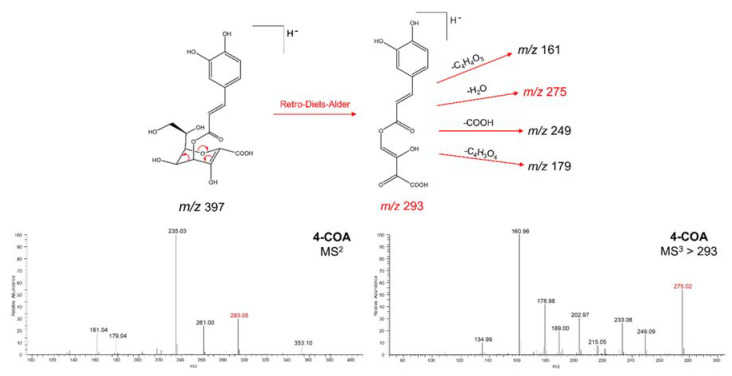
Proposed fragmentation pattern of compound **1** and its mass spectra (MS^2^ and MS^3^) in the negative ionization mode.

To test whether the different isomers of monocaffeoyl-2,7-anhydro-2-octulopyranosonic acid can be distinguished by mass spectrometry alone, we submitted compounds **1** and **2** to tandem mass spectrometry analyses in an ion trap mass spectrometer (see Methods). From this analysis, we found important differences in ion intensities in the MS^2^ and MS^3^ spectra of compounds **1** and **2** (Figure 3 and Figure 4) resulting from differences in the stability of characteristic fragment ions. The main difference lies in the difference in intensity of the ions at *m/z* 293 (MS^2^) and *m/z* 275 (MS^3^) for each of the isomers (Figure 3 and Figure 4). In 5-*O*-caffeoyl-2,7-anhydro-d-glycero-β-d-galacto-oct-2-ulopyranosonic acid (compound **2**), the ion at *m/z* 293 showed an intensity superior to 80% (Figure 3), while in 4-*O*-caffeoyl-2,7-anhydro-d-glycero-β-d-galacto-oct-2-ulopyranosonic acid (compound **1**), the intensity of the same ion was below 50% (Figure 4). These intensity differences were replicated over several repeats. We believe the differences in the intensity of this characteristic fragment ion are due to different fragmentation mechanisms favored by the presence of a free or esterified hydroxyl group in position C4. While in compound **2,** the ion at *m/z* 293 is likely formed by a remote hydrogen rearrangement of the proton of the hydroxyl group at C4 (Figure 3), in compound **1**, this ion is likely formed by a Retro-Diels-Alder reaction in the 2,7-anhydro-2-octulopyranosonic acid moiety (Figure 4). Interestingly, the ion at *m/z* 275 (MS^3^), formed by an H_2_O elimination from *m/z* 293, followed the inverse tendency, with a relative intensity below 50% in compound **2** (Figure 3) and above 50% in compound **1** (Figure 4). These results suggest that, similar to the different isomers of mono- and dicaffeoylquinic acid [22], isomeric caffeoyloctulosonic acids (compounds **1** and **2**) can be distinguished based on their characteristic MS^2^ and MS^3^ spectra, which will aid in future dereplication studies. 

Compounds **5**–**9** were identified as caffeoylaltraric acids by the analysis of their NMR, HRMS, and MS/MS spectra. Compound **9** was identified as 2,3,5- or 2,4,5-tricaffeoylaltraric acid methyl ester. This compound, previously reported only from *Galinsoga parviflora* (Asteraceae) [23], showed a deprotonated molecule, [M − H]^−^, at *m/z* 709.14075 (Figure S6), consistent with the molecular formula of C_34_H_30_O_17_ (calculated for C_34_H_29_O_17_; *m/z* 709.14102). The MS^2^ spectrum of **9** (Figure S7) showed a base peak at *m/z* 547 and a fragment ion at *m/z* 385, consistent with a neutral loss of one and two caffeoyl moieties, respectively (Figure S7). The MS^3^ spectrum of **9** (Figure S8) showed a base peak at *m/z* 353, consistent with a neutral loss of a methanol unit and a fragment ion at *m/z* 223, suggesting that this compound possess three caffeic acid units esterified to a methoxylated core of altraric acid. The ^1^H NMR spectra of **9** (Figure S9) was similar to the ^1^H NMR spectra of compound **5** (2,3,5- or 2,4,5-tricaffeoylaltraric acid) [9], but an additional 3H singlet at *δ* 3.8 ppm, consistent with a methoxy group (Table S1), was observed. The ^13^C spectra (Figure S10) included a shift in the methoxy region (53.04 ppm), which demonstrated a coupling in HSQC to the 3H singlet at 3.8 ppm (Figure S11). Both ^1^H and ^13^C NMR spectra of **9** are in accordance with [23]. Long-range coupling of the same singlet in HMBC (Figure S12) to the altraric acid carbon at 169.26 ppm confirmed the presence of the methoxy group at position C1 or C6, attached to the biester side of the molecule (Table S1). On the other hand, compound **8** was identified as 2,4- or 3,5-dicaffeoylaltraric acid [9], while compounds **6** and **7** were identified as 2- or 5-caffeoylaltraric acid and 3- or 4-caffeoylaltraric acid, respectively, based on the comparison of their ^1^H NMR (Table S2) spectra with the dicaffeoyl derivatives previously reported in yacon [9]. To the best of our knowledge, this study is the first to report compound **9** in a species of the genus *Smallanthus*, and to confirm the presence of compounds **6** and **7** in yacon by their isolation and structural elucidation by NMR.

An attempt to isolate the other tricaffeoylaltraric acid analogs suggested by the FBMN analysis (metabolites with *m/z* values of 679.1309 and 723.1573; Figure 1) was unsuccessful, given their very low concentrations in the roots of yacon.

Lastly, compounds **4** and **10**–**12** were identified as different isomers of mono- and dicaffeoylquinic acids. While compound **4** was identified as 5-*O*-(*E*)-caffeoylquinic acid by the comparison of its NMR and MS/MS spectra with literature information [22,24], compound **10** was suggested as 3-*O*-(*E*)-caffeoylquinic acid by the interpretation of its MS^2^ spectra and its comparison with literature information. This compound showed a deprotonated molecule, [M − H]^−^, at *m/z* 353. The presence of a base peak at *m/z* 191 in the MS^2^ spectra of **10** and a characteristic fragment ion at *m/z* 179 with a relative intensity of ca. 50% allowed its differentiation from the other monocaffeoylquinic acid isomers esterified at positions C1, C4, and C5 [22]. Similarly, compounds **11** and **12** were suggested as 3,5-di-*O*-(*E*)-caffeoylquinic acid and 1,5-di-*O*-(*E*)-caffeoylquinic acid, respectively. These two dicaffeoylquinic acid isomers were distinguished by the intensity of the fragment ion at *m/z* 179, which is superior to the 40% in compound **11**, while in compound **12,** this ion was below 10%, in accordance with a previous study [22].

## 3. Materials and Methods 

### 3.1. Plant Material

An adult plant of *Smallanthus sonchifolius* was collected from the living collection of the Royal Botanic Gardens, Kew. Different organs, including the leaves, stems, roots, bracts, and ray flowers, were separated manually, freeze-dried, and ground. The stem epidermis and attached cortex cells (termed outer stems) were manually separated from the inner parts (vascular tissues) and analyzed independently.

### 3.2. Extraction of Metabolites and UHPLC-UV-HRMS/MS Analysis

Each of the powdered plant parts (leaves 130 g, inner stems 92 g, roots 95 g, outer stems 72 g, and bracts and ray flowers ca. 480 g) were initially extracted with dichloromethane and then with methanol 80% in a 1:10 plant/solvent ratio (g/mL) for 24 h in two consecutive steps with each solvent. Solvents were evaporated to dryness, and the aqueous–methanolic extracts were then lyophilized to yield dried extracts of leaves (35 g), inner stems (30 g), roots (22 g), outer stems (16 g), and bracts and ray flowers (50 mg).

For LC-MS analysis, 10 mg of each of the lyophilized methanolic extracts were redissolved in 1 mL of HPLC-grade methanol 50% by vortexing for a few seconds, followed by ultrasonication at room temperature for 10 min at 40 kHz. Metabolic profiling of caffeic acid esters was performed by UHPLC-UV-HRMS/MS on a Vanquish UHPLC system (Thermo Scientific, Waltham, MA, USA) coupled to a 100 Hz photodiode array detector (PDA) and an Orbitrap Fusion Tribrid (Thermo Scientific) high-resolution tandem mass spectrometer.

Chromatographic separation of plant extracts (5 µL) was performed on a Luna C18 column (150 mm × 3 mm i.d., 3 μm, Phenomenex, Torrance, CA, USA) using a mobile phase gradient of 0:90:10 to 90:0:10 (MeOH (A): water (C): acetonitrile +1% formic acid (D)) over 60 min. Then, 90% A was held for 10 min and then returned to initial conditions over 5 min at 30 °C (flow rate: 400 μL/min). UV detection was recorded between 210 and 550 nm.

Mass spectrometry detection was performed in both positive and negative ionization modes using the full scan and data-dependent MS^2^ and MS^3^ acquisition modes. Total ion current (TIC) chromatograms were obtained over the range of 125–1800 *m/z* using a spray voltage of +3.5 and −2.5 kV for the positive and negative ionization modes, respectively. Four different scan events were recorded for each ionization mode as follows: (1) full scan, (2) MS^2^ of the most intense ion in Scan Event 1, (3) MS^3^ of the most intense ion in Scan Event 2, and (4) MS^3^ of the second most intense ion in Scan Event 2. Additional parameters for the mass spectrometer included full scan resolution, 60,000 FWHM; capillary temperature, 350 °C; ion transfer tube temperature, 325 °C; RF lens (%), 50; automatic gain control (AGC) target, 4.0 × 10^5^ (full scan) and 1.0 × 10^4^ (MS^n^); intensity threshold, 1.0 × 10^4^; CID collision energy, 35 eV; activation Q, 0.25; isolation window (*m/z*), 4. Nitrogen was used as the drying, nebulizer, and fragmentation gas.

### 3.3. Feature-Based Molecular Networking

Feature-based molecular networking (FBMN) was created following the workflow by [18] on the GNPS platform (https://gnps.ucsd.edu, [17]). Chromatographic data in raw format of the negative ionization mode were transformed to mzXML format using the MSConvert package from the software ProteoWizard 3.0.9798 (Proteowizard Software Foundation, Palo Alto, CA, USA). The mass spectrometry data were then processed with MZmine 2.53 [25], and the results were exported to GNPS for FBMN analysis. Raw LC-MS data and the detailed parameters used in MZmine (available as an MZmine batch) are freely available in the MassIVE repository (MSV000086127). 

For FBMN, the data were filtered by removing all MS/MS fragment ions within +/−17 Da of the precursor *m/z*. MS/MS spectra were window-filtered by choosing only the top 6 fragment ions in the +/−50 Da window throughout the spectrum. The precursor and fragment ion mass tolerance were both set to 0.05 Da. A molecular network was then created where edges were filtered to have a cosine score above 0.65 and more than 6 matched peaks. Furthermore, edges between two nodes were kept in the network only if each of the nodes appeared in each other’s respective top 10 most similar nodes. Finally, the maximum size of a molecular family was set to 100, and the lowest-scoring edges were removed from molecular families until the molecular family size was below this threshold. The spectra in the network were then searched against GNPS spectral libraries [17,26]. The library spectra were filtered in the same manner as the input data. All matches kept between network spectra, and library spectra were required to have a score above 0.7 and at least 4 matched peaks. The molecular networks were visualized using the software Cytoscape [27].

To confirm and expand the spectral library annotation made by molecular networking, accurate mass values, MS/MS fragment ions, and UV spectra of the detected metabolites were manually inspected and compared with literature data and information available in the Asteraceae database (AsterDB, www.asterbiochem.org/asterdb), which includes all the caffeic acid esters reported in the genus *Smallanthus*. To have an overview of the confidence level achieved in the identification of metabolites, we adopted the four levels of accuracy reported in the Metabolomics Standard Initiative [28]. 

### 3.4. Isolation of Caffeic Acid Esters

A sample of yacon inner stem (10 g) and root (10 g) extracts were independently submitted to Sephadex LH-20 column chromatography (120 g, 400 × 40 mm i.d.) employing mixtures of 300 mL of water–methanol (100:0, 80:20, 60:40, 40:60, 80:20, 0:100). Chromatographic separation of the inner stem extract (10 g) and UHPLC-UV-MS/MS monitoring afforded ten fractions (Fr1–Fr10). Fr 5, Fr 7, and Fr 8 were further purified by semipreparative HPLC (Waters e2695, Waters, Milford, MA, USA) using a Luna C18 column (250 × 10 mm i.d., 10 μm, Phenomenex) and a mixture of MeOH (A):water (C):acetonitrile + 1% formic acid (D) (0:90:10 to 90:0:10) over 20 min. Fr 5 yielded 32 mg of 4-*O*-caffeoyl-2,7-anhydro-d-glycero-β-d-galacto-oct-2-ulopyranosonic acid (**1**) [10] and 2 mg of the new 5-*O*-caffeoyl-2,7-anhydro-d-glycero-β-d-galacto-oct-2-ulopyranosonic acid (**2**) (Table 1). Fr 7 afforded 10 mg of 5-*O*-(*E*)-caffeoylquinic acid (**4**) [24], while Fr 8 yielded 12 mg of 4,5-di-*O*-caffeoyl-2,7-anhydro-d-glycero-β-d-galacto-oct-2-ulopyranosonic acid (**3**) [10]. Fr 10 (46 mg) was constituted by pure 2,3,5- or 2,4,5-tricaffeoylaltraric acid (**5**) [9]. Chromatographic separation of the root extract (10 g) afforded seven fractions (RFr1–RFr7), of which Fr 2–4, and Fr 7 were purified by semipreparative HPLC. RFr 2 yielded 1 mg of 2- or 5-caffeoylaltraric acid (**6**) and 3 mg of 3- or 4-caffeoylaltraric acid (**7**) [9]. RFr 3 afforded 50 mg of 2,4- or 2,5-dicaffeoylaltraric acid (**8**) [9], while RFr 4 yielded additional 130 mg of 2,3,5- or 2,4,5-tricaffeoylaltraric acid (**5**) [9]. Lastly, semipreparative purification of RFr 7 afforded 2.5 mg of 2,3,5- or 2,4,5-tricaffeoylaltraric acid methyl ester (**9**; Table S1). The structures of the isolated metabolites (Figure 2) were identified by uni- and bidimensional NMR experiments (Bruker ARX 400, Billerica, MA, USA, MeOD) and by high-resolution MS (Orbitrap Fusion, Thermo Scientific) and low-resolution tandem mass spectrometry (Ion Trap Velos Pro, Thermo Scientific).

## Figures and Tables

**Table 1:** NMR shifts (ppm) of compounds **1** (4-*O*-caffeoyl-2,7-anhydro-d-glycero-β-d-galacto-oct-2-ulopyranosonic acid, 4-COA), **2** (5-*O*-caffeoyl-2,7-anhydro-d-glycero-β-d-galacto-oct-2-ulopyranosonic acid, 5-COA), and **3** (4,5-di-O-caffeoyl-2,7-anhydro-d-glycero-β-d-galacto-oct-2-ulopyranosonic acid, 4,5-COA).

	Compound 2 (5-COA)			Compound 1 (4-COA)		Compound 3 (4,5-diCOA)	
	^13^C	^1^H	HMBC	^13^C	^1^H	^13^C	^1^H
1	170.3	-	-	170.6	-	170.2	-
2	105.3	-	-	104.9	-	115.1	-
3	75.1	4.07, bs	4,5	73.0	4.05, br s	73.6	4.12, brs
4	70.7	4.16, bd (5.7)	2,6,3,5	73.3	5.27 d (5.3)	71.1	5.43, d (6.3)
5	69.3	5.30, dd (5.7, 4.2)	1’,11,12,13	65.4	4.34 dd (4.4, 5.3)	66.6	5.56, dd (4.7, 6.3)
6	76.6	4.66, dd (4.2, 3.9)	2,4	78.8	4.46, dd (4.4, 3.7)	76.2	4.70, dd (4.7, 3.7)
7	82.4	4.27, ddd (3.9, 4.7, 8.2)	8	82.5	4.28, ddd (3.7, 4.2, 8.2)	82.3	4.34, ddd (3.7, 4.2, 7.8)
8	60.6	4.09, dd (4.7, 11.8)	2	60.8	4.37 dd (8.2, 12.1)	60.7	4.40, dd (7.8, 11.4)
		4.37, dd (8.2, 11.8)	7,6		4.04, dd (4.2, 12.1)		4.06, dd (4.2, 11.4)
**4-caffeoyl**							
1′	-	-	-	168.4	-	167.8	-
2′	-	-	-	115.2	6.36, d (15.9)	114.4	6.36, d (15.9)
3′	-	-	-	147.4	7.63, d (15.9)	148.4	7.63, d (15.9)
1″	-	-	-	127.8	-	127.6	-
2″	-	-	-	115.2	7.09, d (1.9)	115.3	7.09, d (2.1)
3″	-	-	-	146.8	-	149.9	-
4″	-	-	-	149.6	-	149.9	-
5″	-	-	-	116.5	6.78, d (8.2)	116.5	6.77, d (8.1)
6″	-	-	-	123.1	6.99, dd (1.9, 8.2)	123.5	6.99, dd (2.1, 8.1)
**5-caffeoyl**							
1′	167.9	-	-	-	-	167.1	-
2′	114.6	6.32, d (15.9)	1′,1″	-	-	113.9	6.14, d (15.9)
3′	147.7	7.62, d (15.9)	1′,1″,2″,2′6″	-	-	148.1	7.46, d (15.9)
1″	127.7	-	-	-	-	127.4	-
2″	115.2	7.06, d (2.0)	4″,6″	-	-	114.8	6.98, d (2.1)
3″	146.8	-	-	-	-	146.9	-
4″	149.7	-	-	-	-	149.9	-
5″	116.5	6.78, d (8.1)	1″,3″	-	-	116.4	6.65, d (8.2)
6″	123.1	6.97, dd (2.0, 8.1)	2″,3′	-	-	123.7	6.77, dd (2.1, 8.2)

## Supplementary Materials

The following are available online at https://www.mdpi.com/2218-1989/10/10/407/s1. Figure S1: ^1^H NMR spectrum of compound **2**, Figure S2: ^13^C NMR spectrum of compound **2**, Figure S3: HSQC spectrum of compound **2,** Figure S4: HMBC of compound mixture of **1** and **2**, Figure S5: HRMS spectrum of compound **2**, Figure S6: HRMS spectrum of compound **9**, Figure S7: MS^2^ spectrum of compound **9**, Figure S8: MS^3^ spectrum of ion at 385 *m/z* in MS^2^, compound **9**, Figure S9: ^1^H NMR spectrum of compound **9**, Figure S10: ^13^C NMR spectrum of compound **9**, Figure S11: HSQC NMR spectrum of compound **9**, Figure S12: HMBC NMR spectrum of compound **9**, Table S1: NMR shifts (ppm) of 2,3,5- or 2,4,5-tricaffeoylaltraric acid methyl ester (compound **9**), Table S2. 1H NMR shifts (ppm) of compounds **5** (2,3,5- or 2,4,5-triCAA), **6** (2- or 5-mCAA), **7** (3- or 4-mCAA) and **8** (2,4- or 3,5-diCAA).
