# Supplementary material for: Feature-Based Molecular Networking to Target the Isolation of New Caffeic Acid Esters from Yacon (Smallanthus sonchifolius, Asteraceae)
Source: Metabolites. 2020 Oct 13;10(10):407. doi: 10.3390/metabo10100407 (PMC7601859; doi:10.3390/metabo10100407)
Supplement: Supplementary file 1 [file metabolites-10-00407-s001.pdf]

Feature-based molecular networking to target the isolation of new caffeic acid esters from yacon (*Smallanthus sonchifolius*, Asteraceae)

Guillermo F. Padilla-González[1], Nicholas J. Sadgrove[1], Gari V. Ccana-Ccapatinta[2], Olga Leuner[3], Eloy Fernandez-Cusimamani[3,*]

[1] Jodrell Laboratory, Royal Botanic Gardens, Kew, Kew Green Road, TW9 3AB, London, United Kingdom; f.padilla@kew.org (GFP-G); n.sadgrove@kew.org (NJS).

[2] AsterBioChem Research Team, Laboratory of Pharmacognosy, School of Pharmaceutical Sciences of Ribeirão Preto, University of São Paulo, Av do café s/n, 14040-903 Ribeirão Preto, SP, Brazil; febcosta@fcfrp.usp.br; cuscogarig@hotmail.com

[3] Czech University of Life Sciences Prague, Faculty of Tropical AgriSciences, Department of Crop Sciences and Agroforestry, Kamýcká 129, 165 00, Prague, Czech Republic; eloy@ftz.czu.cz (EFC); leuner@ftz.czu.cz (OL).

* Correspondence: eloy@ftz.czu.cz (EFC) and f.padilla@kew.org (GFPG).

Supplementary Information

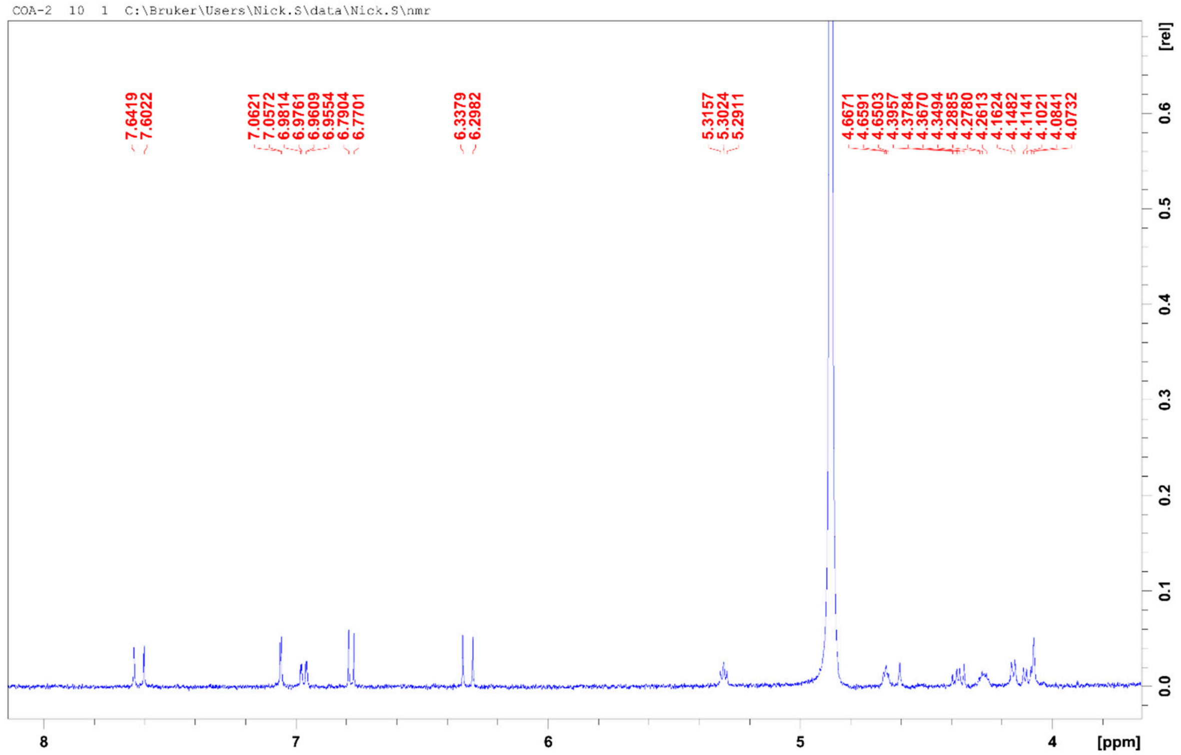

Figure S1. ^{1}H NMR spectrum of compound **2**.

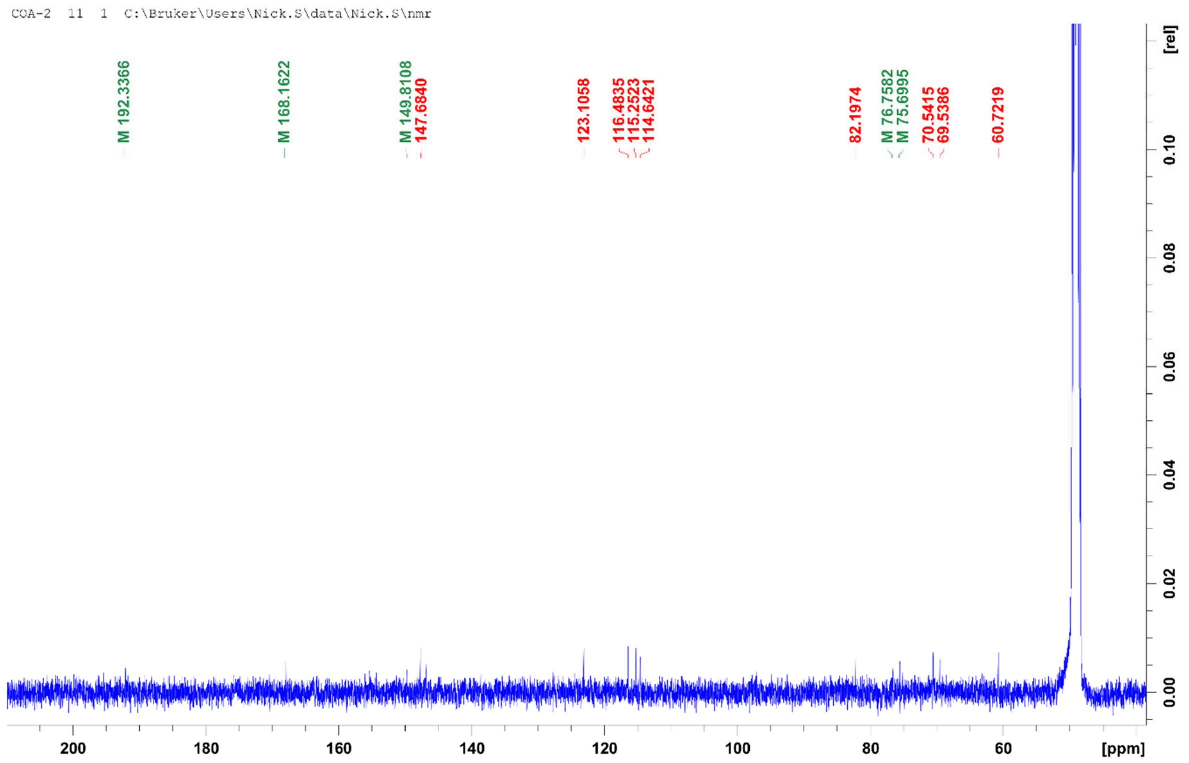

Figure S2. ^{13}C NMR spectrum of compound **2**.

Figure S3. HSQC spectrum of compound **2**

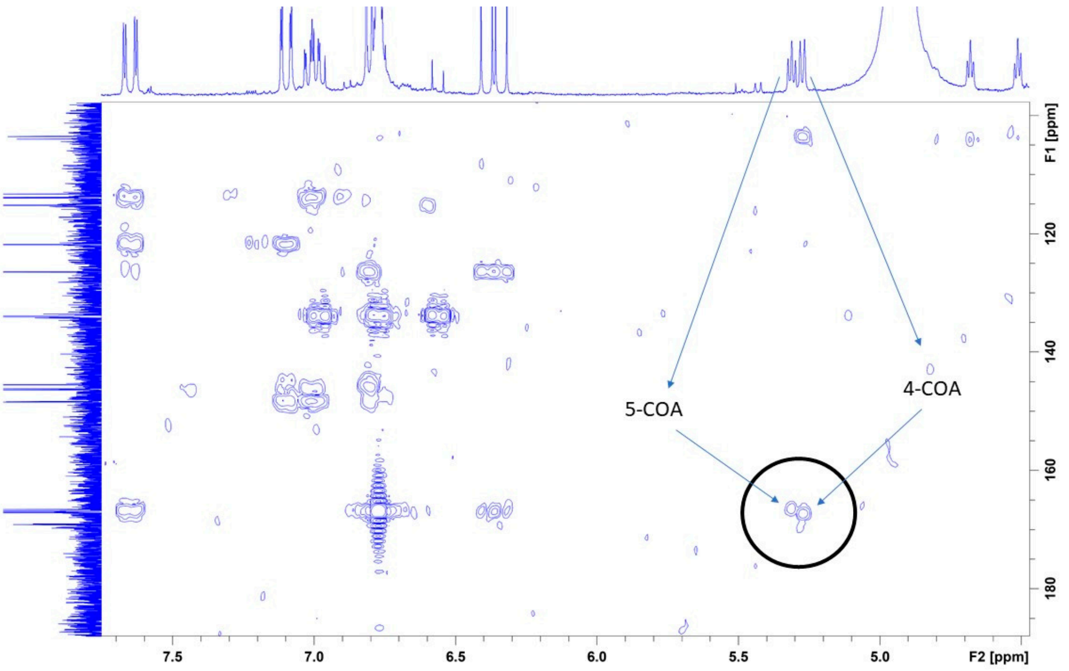

Figure S4. HMBC of compound mixture of **1** and **2**. The HMBC of the pure compound **2** was not resolved to convey the highlighted correlation here. The circled couplings are for the caffeoyl carbonyl carbon to the hydroxy-proton of the adjoining ester link.

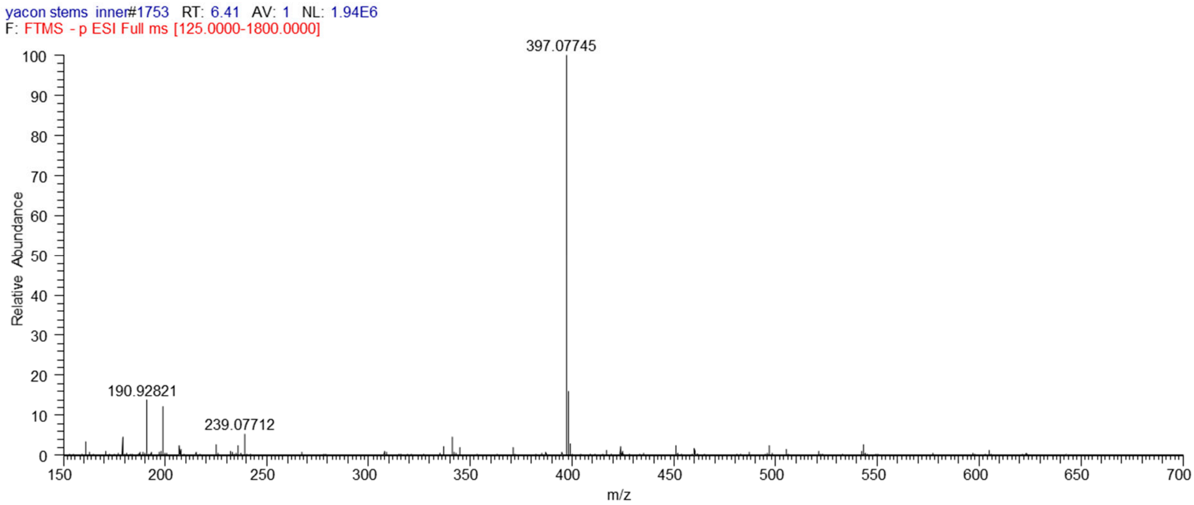

Figure S5. HRMS spectrum of compound **2**.

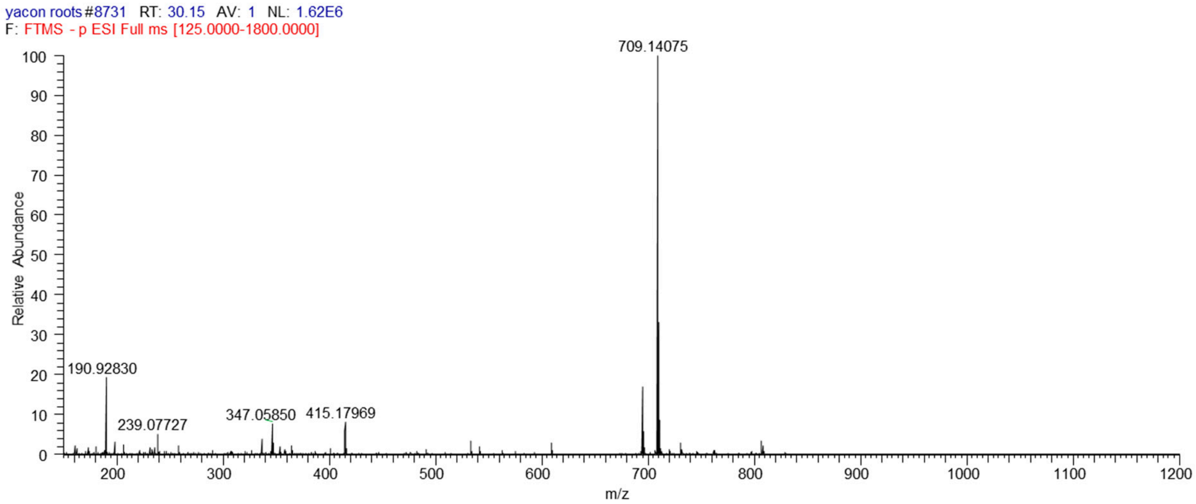

Figure S6. HRMS spectrum of compound **9**.

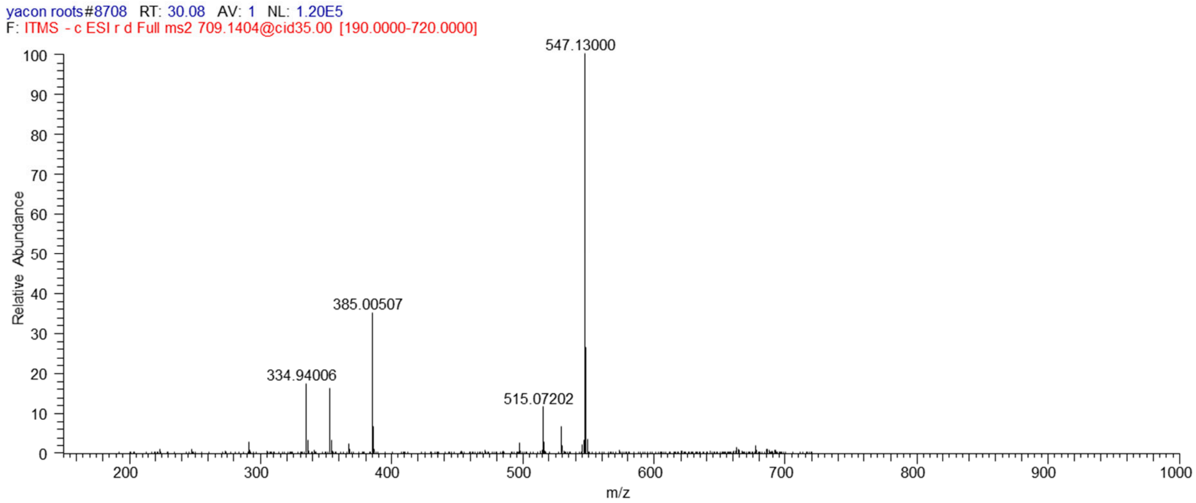

Figure S7. M^2 spectrum of compound **9**.

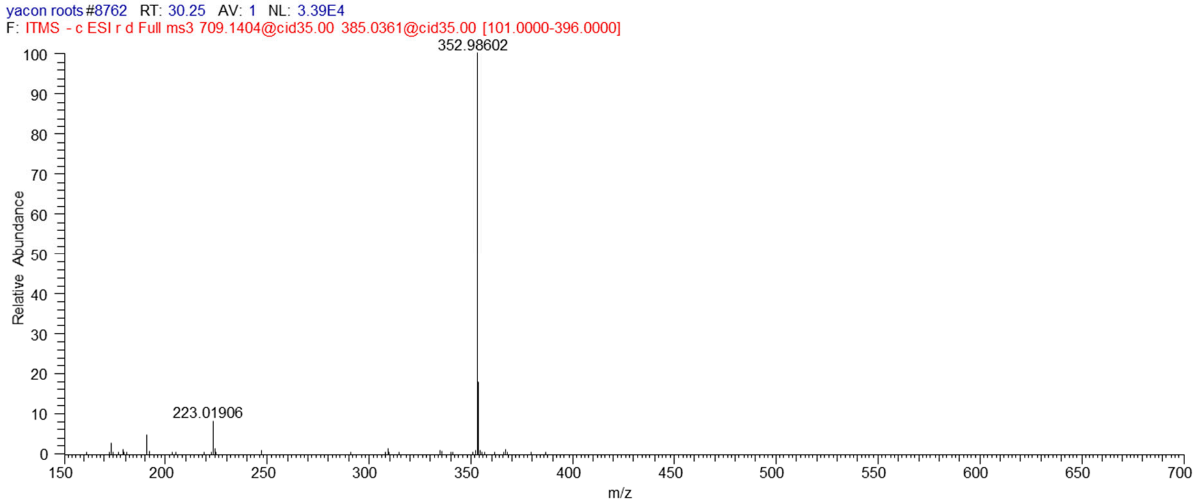

Figure S8. M^3 spectrum of ion at 385 *m/z* in MS^2, compound **9**.

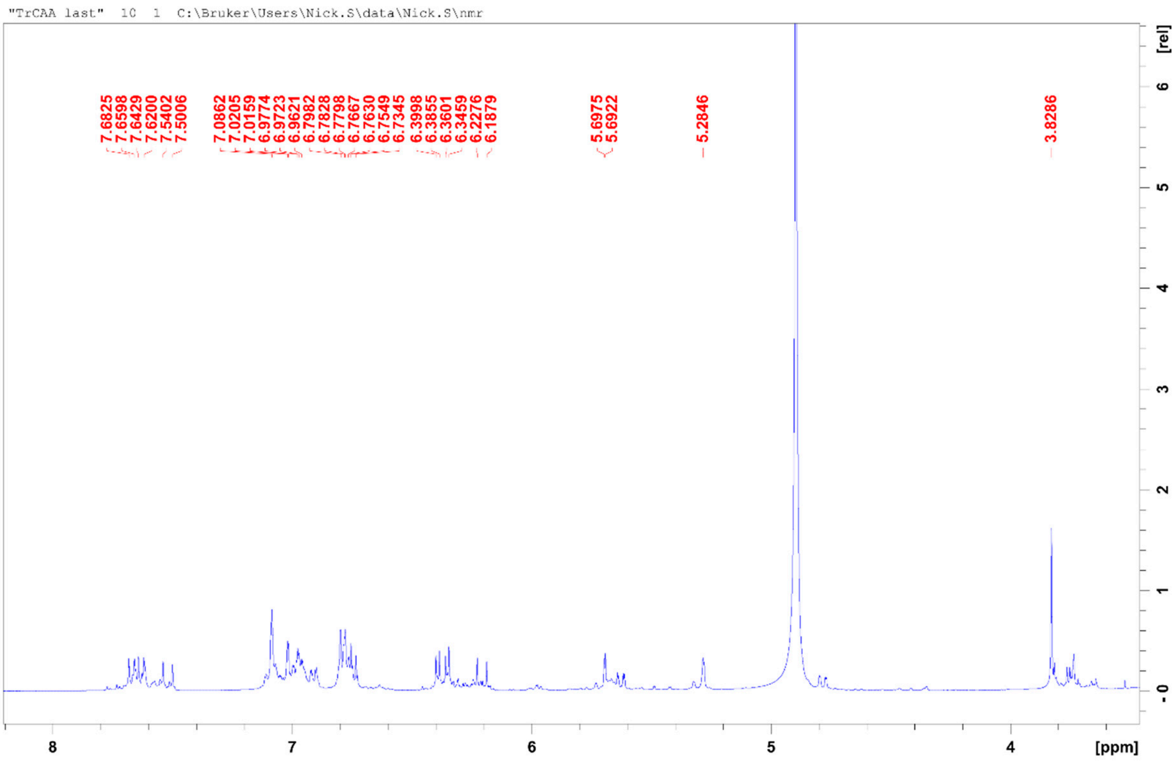

Figure S9. ^{1}H NMR spectrum of compound **9**.

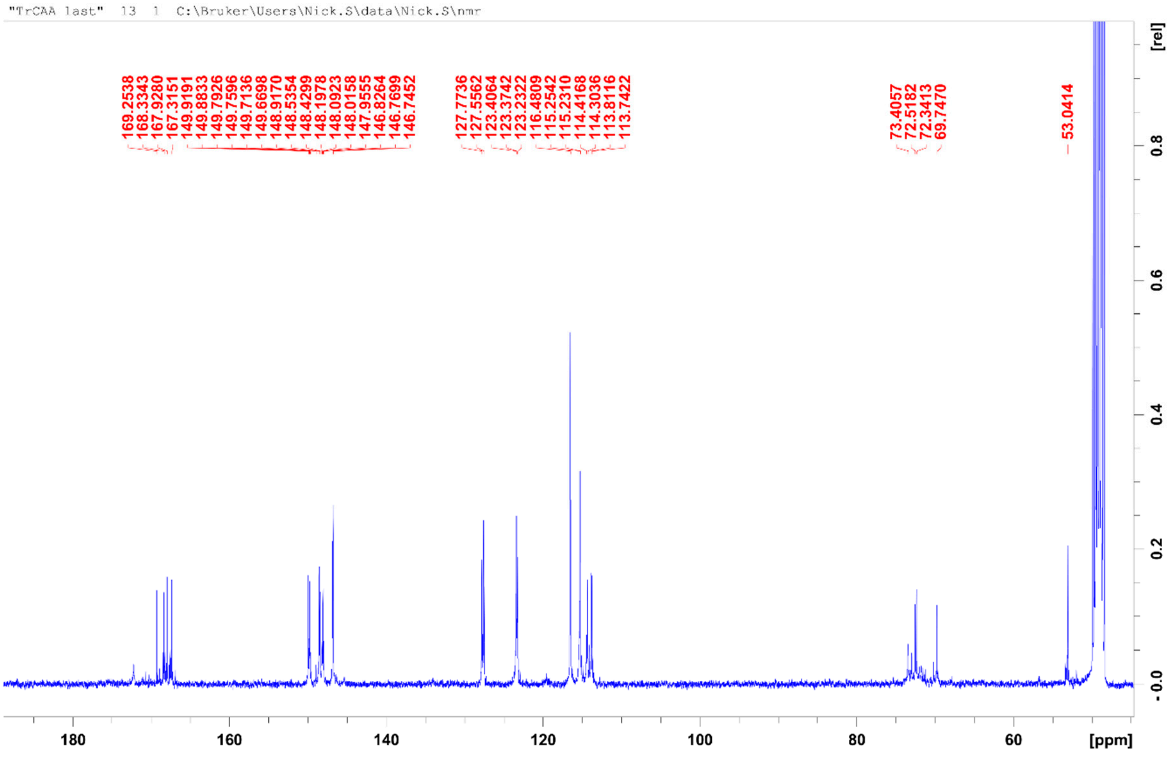

Figure S10. ^{13}C NMR spectrum of compound **9**.

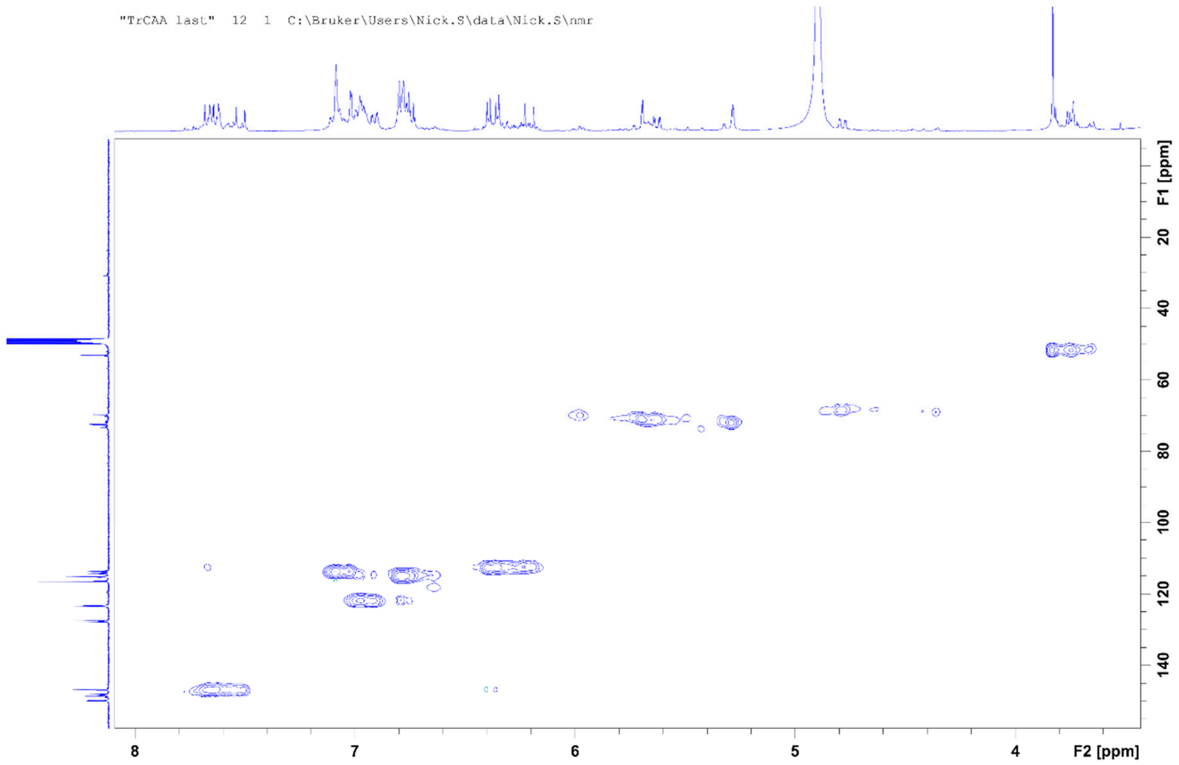

Figure S11. HSQC NMR spectrum of compound **9**.

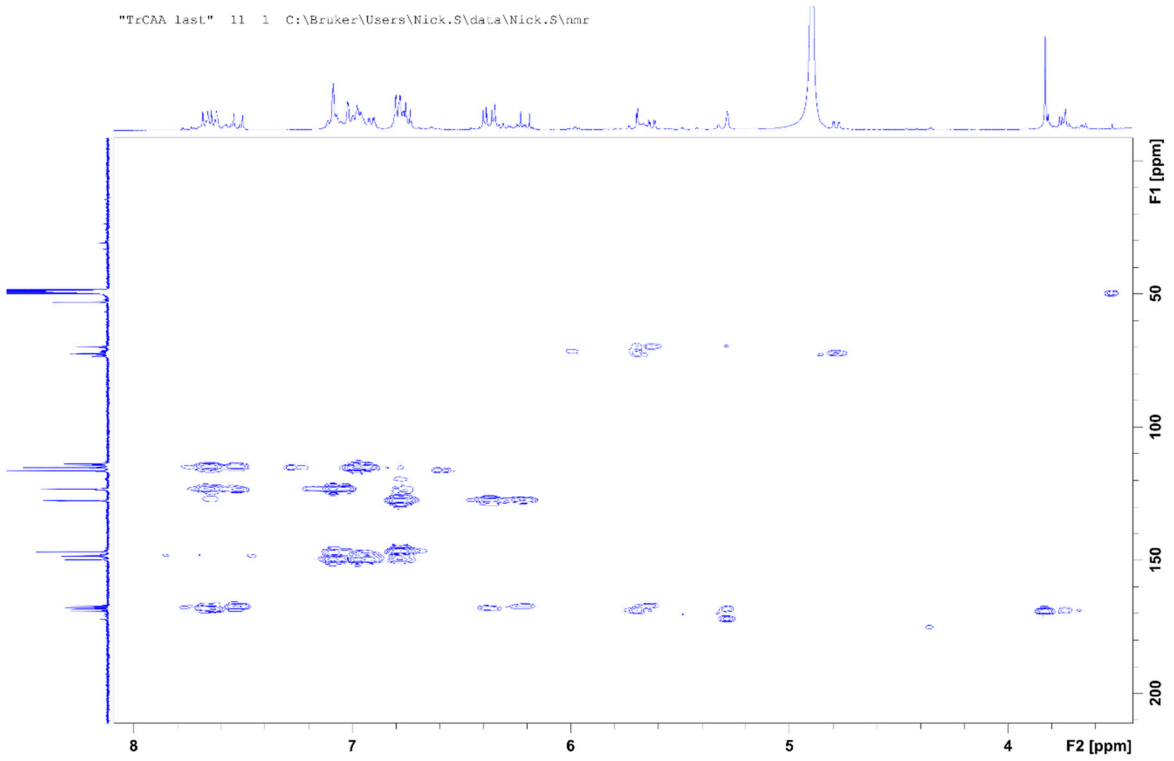

Figure S12. HMBC NMR spectrum of compound **9**.

Table S1. NMR shifts (ppm) of 2,3,5- or 2,4,5-tricaffeoylaltraric acid methyl ester (compound **9**).

**Altraric acid methyl ester**			**2(5)-*O*-caffeoyl'**		
	**^{13}C**	**^{1}H**		**^{13}C**	**^{1}H**
1(6)	172.17	-	1′	127.78	-
2(5)	73.41	5.28, d, 1.9 Hz	2′	115.26	7.09, d, 1.8 Hz
3(4)	69.75	4.78, dd, 9.9, 1.9 Hz	3′	146.77	-
4(3)	72.34	5.63, dd, 2.2, 9.9 Hz	4′	149.72	-
5(2)	72.53	5.70, d, 2.2 Hz	5′	116.49	6.79, d, 8.2 Hz
6(1)	169.26	-	6′	123.38	6.99, dd, 2.0, 8.2 Hz
O-CH_3	53.04	3.83, 3Hs	7′	148.54	7.64, d, 15.9 Hz
			8′	114.31	6.37, d, 15.9 Hz
			9′	168.34	-
**3(4)-*O*-caffeoyl′′**			**5(2)-*O*-caffeoyl′′′**		
1′′	127.47	-	1′′′	127.56	-
2′′	115.23	7.02, d, 1.8 Hz	2′′′	115.19	7.09, d, 1.8 Hz
3′′	146.83	-	3′′′	146.75	-
4′′	149.89	-	4′′′	149.93	-
5′′	116.51	6.74, d, 8.2 Hz	5′′′	116.5	6.79, d, 8.2 Hz
6′′	123.42	6.91, dd, 2.0, 8.2 Hz	6′′′	123.24	6.97, dd, 2.0, 8.2 Hz
7′′	148.43	7.52, d, 15.9 Hz	7′′′	148.02	7.66, d, 15.9 Hz
8′′	113.74	6.21, d, 15.9 Hz	8′′′	113.81	6.38, d, 15.9 Hz
9′′	167.32	-	9′′′	167.93	-

Table S2. 1H NMR shifts (ppm) of compounds **5** (2,3,5- or 2,4,5-triCAA), **6** (2- or 5-mCAA), **7** (3- or 4-mCAA) and **8** (2,4- or 3,5-diCAA).

	**2,3,5- or 2,4,5-triCAA**		**2,4- or 3,5-diCAA**		**2- or 5-mCAA** (MeOD:D2O)		**3- or 4-mCAA** (MeOD:D2O)	
**altraric acid**	*δ* (ppm)	*J* (Hz)	*δ* (ppm)	*J* (Hz)	*δ* (ppm)	*J* (Hz)	*δ* (ppm)	*J* (Hz)
2(5)	5.68, d	1.8	5.25, d	1.8	5.3, d	1.9, d	4.35, d	1.9
3(4)	5.66, dd	1.8, 9.9	4.77, dd	1.8, 9.8	4.37, dd	1.9, 8.4, dd	5.40, dd	1.9, 9.2
4(3)	4.84, dd	9.9, 1.8	5.49, d	9.8	3.93, dd	8.4, 1.9, dd	4.38, dd	9.2, 1.3
5(2)	5.29, d	1.8	4.54, brs	-	4.16, d	1.9, d	3.99, d	1.3
2(5)-O-caffeoyl	-	-	-	-	-	-	-	-
2	4.09, d	1.9	7.08, d	2.1	7.13, d	2.1	-	-
5	6.78, d	8.1	6.78, d	8.3	6.82, d	8.2	-	-
6	6.97, dd	1.9, 8.1	6.96, dd	2.1, 8.3	7.01, dd	2.1, 8.2	-	-
7	7.66, d	15.9	7.64, d	15.9	7.65, d	16.0	-	-
8	6.37, d	15.9	6.35, d	15.9	6.46, d	16.0	-	-
3(4)-O-caffeoyl	-	-	-	-	-	-	-	-
2	7.02, d	1.9	-	-	-	-	7.15, d	2.1
5	6.74, d	8.1	-	-	-	-	6.87, d	8.2
6	6.91, dd	1.9, 8.1	-	-	-	-	7.07, dd	2.1, 8.2

7	7.53, d	15.9	-	-	-	-	7.66, d	15.9
8	6.22, d	15.9	-	-	-	-	6.39, d	15.9
4(3)-O-caffeoyl	-	-	-	-	-	-	-	-
2	-	-	7.02, d	2.0	-	-	-	-
5	-	-	6.76, d	8.3	-	-	-	-
6	-	-	6.92, dd	2.0, 8.3	-	-	-	-
7	-	-	7.53, d	15.8	-	-	-	-
8	-	-	6.23, d	15.8	-	-	-	-
5(2)-O-caffeoyl	-	-	-	-	-	-	-	-
2	7.07, d	1.9	-	-	-	-	-	-
5	6.78, d	8.2	-	-	-	-	-	-
6	6.97, dd	1.9, 8.2	-	-	-	-	-	-
7	7.64, d	15.8	-	-	-	-	-	-
8	6.36, d	15.8	-	-	-	-	-	-
